# A systematic review and meta-analysis of enrollment into ARDS and sepsis trials published between 2009 and 2019 in major journals

**DOI:** 10.1186/s13054-021-03804-1

**Published:** 2021-11-15

**Authors:** Dustin C. Krutsinger, Kuldeep N. Yadav, Michael O. Harhay, Karsten Bartels, Katherine R. Courtright

**Affiliations:** 1 Division of Pulmonary, Critical Care, and Sleep Medicine, University of Nebraska Medical Center, 985910 NE Medical Center, Omaha, NE 68198 USA; 2 Palliative and Advanced Illness Research (PAIR) Center, University of Pennsylvania, 300 Blockley Hall, 423 Guardian Drive, Philadelphia, PA 19104 USA; 3 Department of Biostatistics, Epidemiology, and Informatics, Perelman School of Medicine, University of Pennsylvania, Philadelphia, PA USA; 4 Pulmonary, Allergy, and Critical Care Division, Perelman School of Medicine, University of Pennsylvania, Philadelphia, PA USA; 5 Department of Anesthesiology, University of Nebraska Medical Center, 985910 NE Medical Center, Omaha, NE 68198 USA

**Keywords:** ARDS, Sepsis, Acute lung injury, Randomized controlled trial, Enrollment

## Abstract

**Background:**

Enrollment problems are common among randomized controlled trials conducted in the ICU. However, little is known about actual trial enrollment rates and influential factors. We set out to determine the overall enrollment rate in recent randomized controlled trials (RCTs) of patients with acute respiratory distress syndrome (ARDS), acute lung injury (ALI), or sepsis, and which factors influenced enrollment rate.

**Methods:**

We conducted a systematic review by searching Pubmed using predefined terms for ARDS/ALI and sepsis to identify individually RCTs published among the seven highest impact general medicine and seven highest impact critical care journals between 2009 and 2019. Cluster randomized trials were excluded. Data were extracted by two independent reviewers using an electronic database management system. We conducted a random-effects meta-analysis of the eligible trials for the primary outcome of enrollment rate by time and site.

**Results:**

Out of 457 articles identified, 94 trials met inclusion criteria. Trials most commonly evaluated pharmaceutical interventions (53%), were non-industry funded (78%), and required prospective informed consent (81%). The overall mean enrollment rate was 0.83 (95% confidence interval: 0.57–1.21) participants per month per site. Enrollment in ARDS/ALI and sepsis trials were 0.48 (95% CI 0.32–0.70) and 0.98 (95% CI 0.62–1.56) respectively. The enrollment rate was significantly higher for single-center trials (4.86; 95% CI 2.49–9.51) than multicenter trials (0.52; 95% CI 0.41–0.66). Of the 36 trials that enrolled < 95% of the target sample size, 8 (22%) reported slow enrollment as the reason.

**Conclusions:**

In this systematic review and meta-analysis, recent ARDS/ALI and sepsis clinical trials had an overall enrollment rate of less than 1 participant per site per month. Novel approaches to improve critical care trial enrollment efficiency are needed to facilitate the translation of best evidence into practice.

**Supplementary Information:**

The online version contains supplementary material available at 10.1186/s13054-021-03804-1.

## Background

Enrollment problems in randomized controlled trials (RCTs) lead to reduced precision regarding interventions’ effects [1], increased study costs [2], and slows the adoption of evidence into practice [3]. Research involving critically ill patients poses unique challenges to trial enrollment, including narrow recruitment windows and reliance on surrogate decision-makers for obtaining informed consent [4, 5]. Moreover, critical care trials are often underpowered to detect a clinically meaningful difference in mortality due to, at least in part, enrollment difficulties [6] Additionally, several recently published high profile critical care trials were terminated early due to difficulties with enrollment [7–9].

Underpowered trials, and those terminated strictly due to feasibility issues, raise ethical concerns as they expose participants to any risks involved in participation with a reduced likelihood of social benefit [10]. In an effort to address issues of trial inefficiencies, it has been proposed that alternative recruitment strategies [11] and trial designs [12] are needed. Accurate estimation of enrollment rate is essential to inform trial planning and to serve as a benchmark for evaluating the utility of novel trial designs. However, reporting of trial recruitment and enrollment metrics varies, and little is known about trial factors that influence the enrollment rate.

We conducted a systematic review of trials that evaluated interventions for common critical care syndromes, including acute respiratory distress syndrome (ARDS), acute lung injury (ALI), and sepsis, to assess enrollment rate and contributing trial design factors. We focused this review on trials published in the last decade to ensure the results reflect contemporary critical care science and recruitment practices to best inform trial design and conduct in the near future.

## Methods

We conducted a systematic review and meta-analysis, following the Preferred Reporting Items for Systematic Reviews and Meta-Analyses guidelines (Additional file 1: Appendix A) [13]. The protocol was not eligible for registration in the International Prospective Register of Systematic Reviews since the outcomes of interest were not health-related [14]. This study did not meet the criteria for human subjects research as defined by United States federal policy 45 Code of Federal Regulations 46 and thus was exempt from review by the institutional review board.

### Data sources

We searched PubMed to identify all ARDS/ALI and sepsis RCTs published from 2009 to 2019 in the seven highest-impact critical care and seven highest impact general medicine journals according to InCites Journal Citation Reports [15]. The following journals met our criteria: (1) The New England Journal of Medicine, (2) Lancet, (3) Journal of the American Medical Association, (4) Nature Reviews Disease Primers, (5) British Medical Journal, (6) JAMA Internal Medicine, (7) Annals of Internal Medicine, (8) Lancet Respiratory Medicine, (9) Intensive Care Medicine, (10) American Journal of Respiratory and Critical Care Medicine, (11) Chest, (12) Critical Care, (13) Critical Care Medicine, and (14) Annals of the American Thoracic Society. Consistent with prior systematic reviews that limited the literature search to high-impact journals when asking questions related to trial design and metrics [6, 16], we restricted our search to high-impact journals in order to increase the likelihood of capturing the highest quality trials and thus are most likely to reflect the “best-case scenario” of enrollment rate. We used the following Medical Subject Headings (MeSH) terms: (1) respiratory distress syndrome, adult, (2) sepsis, and (3) acute lung injury. The full search strategy was determined a priori and is detailed in Additional file 1: Appendix B.

### Study selection and data extraction

Two reviewers (D.C.K., K.N.Y) independently screened abstracts and full-text articles for pre-specified eligibility criteria. Articles were included for full-text review if they were conducted among patients diagnosed with ARDS/ALI or sepsis in the intensive care unit or emergency department, randomized at the patient level, required informed consent, and reported at least one clinical outcome. The risk of bias for trial outcomes was not assessed for this review of trial process metrics.

### Outcomes

The primary outcome was enrollment rate, defined as the number of participants enrolled in each trial per month per site. The secondary outcome was the reported reason that target enrollment (randomized < 95% of target sample size) was not achieved.

### Data synthesis and analysis

Two reviewers (D.C.K., K.N.Y) independently extracted the data from full-text articles using REDCap electronic database [17]. The primary outcome of enrollment rate was calculated by dividing the number of participants enrolled in each trial by the duration of the trial (in months) and then by site. Duration of enrollment was determined from the reported start, and end dates rounded to the nearest month. Trials that did not report a start and stop date for enrollment or did not report the number of participating study sites were not included in the meta-analysis. Study heterogeneity was assessed using Cochran’s *Q* and the *I*^2^ statistic [18]. Due to the significant heterogeneity across studies for the primary outcome, we performed a random-effects meta-analysis to determine the overall enrollment rate using the method of DerSimonian and Laird [19], which adjusts the standard errors of the study estimates to account for study-specific heterogeneity such as sample size.

We conducted a stratified analysis of the primary outcome, stratified by condition ARDS/ALI and sepsis, excluding trials that combined conditions. We conducted the following pre-specified subgroup analyses for the primary outcome: (1) the number of sites (single-center versus multicenter), (2) temporal trend (publication year 2009–2013 versus 2014–2019), (3) funding source (any versus no industry funding), (4) intervention (pharmaceutical versus non-pharmaceutical), (5) continent (first author’s location), (6) informed consent (prospective versus retrospective), and (7) enrollment achieved ($\ge$ 95% versus < 95% of target sample size randomized).

All statistical analyses were performed using the R language for statistical computing (version 4.0.1), and R packages meta (version 4.13–0) and tidyverse (version 1.3.0).

## Results

### Characteristics of included studies

**Fig. 1:**
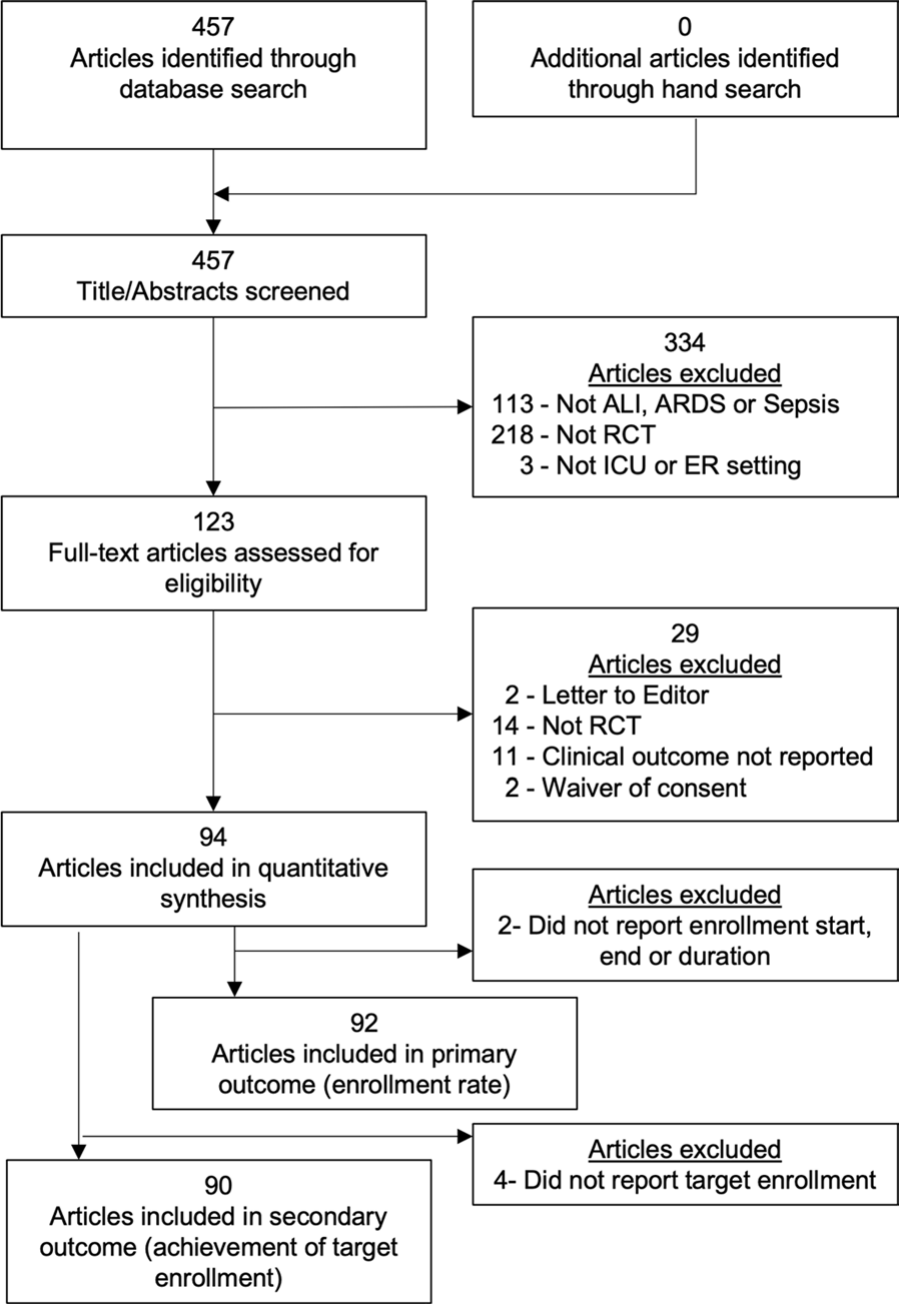
Article selection through the systematic process

Our PubMed search yielded 457 unique articles, of which 94 met eligibility criteria (Fig. 1, Additional file 1: Appendix C). Most trials evaluated pharmaceutical interventions (53%), were non-industry funded (78%), and required prospective informed consent (81%) (Table 1). Recruitment occurred at a median of 18 (IQR 2–38) sites per trial for a median duration of 36 (IQR 24–48) months (Additional file 1: Appendix D).

**Table 1:** Characteristics of articles

Characteristic	Number	Percent
Included studies	94	
*Target patient population*		
Acute respiratory distress syndrome (ARDS)/acute lung injury (ALI)	23	24.5
Sepsis (includes sepsis, severe sepsis, septic shock)	67	71.3
Both sepsis and ARDS	4	4.3
*Publication period*		
2009–2013	43	45.7
2014–2019	51	54.3
*Journal*		
American Journal of Respiratory and Critical Care Medicine	9	9.6
Chest	1	1.1
Critical Care	14	14.9
Critical Care Medicine	16	17.0
Intensive Care Medicine	11	11.7
Journal of the American Medical Association	18	19.1
Journal of the American Medical Association – Internal Medicine	1	1.1
Lancet	2	2.1
Lancet—Respiratory Medicine	2	2.1
New England Journal of Medicine	20	21.3
*Continent of first author*		
Asia	8	8.5
Australia	4	4.3
Europe	46	48.9
North America	21	22.3
South America	15	16.0
*Funding sources*^*a*^		
Academic institution	19	20.2
Association or foundation	14	14.9
Government	47	50.0
Industry	21	22.3
Unreported	9	9.6
*Intervention*^*b*^		
Clinical protocol	32	34.0
Device	12	12.8
Diagnostic test	1	1.1
Drug	50	53.2
*Consent type*		
Prospective	76	80.9
Retrospective	18	19.1
*Enrollment achieved*^*c*^* (N* = *90)*		
$\ge$ 95% of target sample size enrolled	54	60
< 95% of target sample size enrolled	36	40
Futility^a^	15	41.6
Safety^a^	10	27.8
Efficacy^a^	4	11.1
Slow enrollment^a^	8	22.2
Other/not reported^a^	4	11.1

^a^Not mutually exclusive, ^b^One article reported both a pharmacotherapeutic and clinical protocol intervention, ^c^Four trials did not report a sample size target

### Enrollment rate

**Fig. 2:**
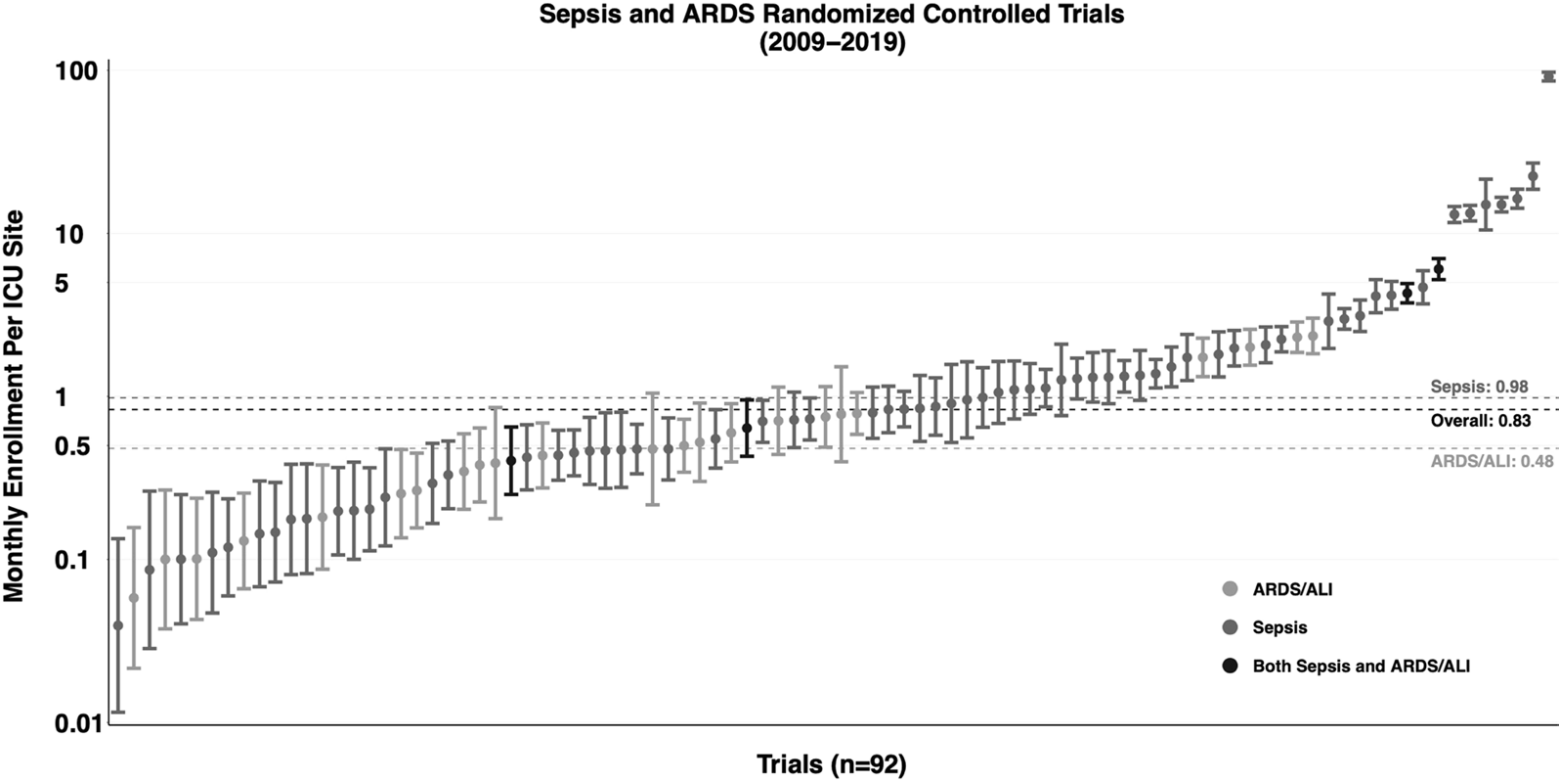
Enrollment rate among ARDS/ALI and sepsis trials published 2009–2019. Enrollment rate defined as the number of participants enrolled per month per site, displayed on log scale. Two trials excluded due to failure to report enrollment duration

**Fig. 3:**
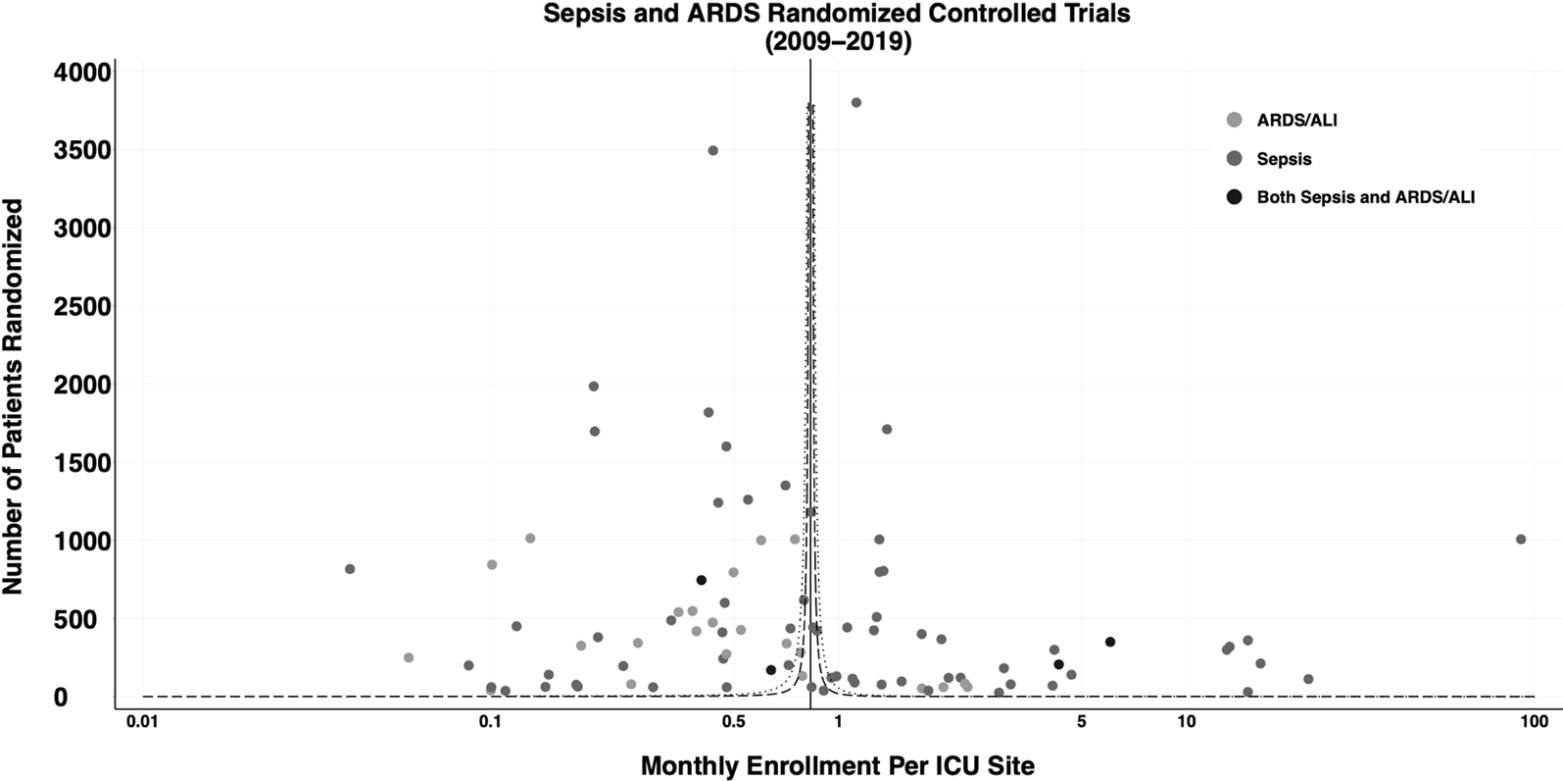
Funnel plot. Number of patients randomized against enrollment rate per month per site, displayed on a log scale. The vertical dotted line represents the overall monthly enrollment rate per site. The two exponential lines represent the 95% (grey, small dashed line) and 99.8% (black, large dashed line) confidence intervals around the overall monthly enrollment rate per site

Two trials did not report their enrollment duration and were excluded from the primary analysis. The overall mean enrollment rate was 0.83 (95% confidence interval [CI] 0.57–1.21) participants per month per site (Fig. 2). ARDS trials had an enrollment rate of 0.48 (95% CI 0.32–0.70), while sepsis trials had an enrollment rate of 0.98 (95% CI 0.62–1.56). Study heterogeneity was assessed using a funnel plot (Fig. 3).

The enrollment rate was significantly higher in single-center (4.86; 95% CI 2.49–9.51) compared to multicenter trials (0.52; 95% CI 0.41–0.66; *p* < 0.0001) and in non-industry funded trials (0.93; 95% CI 0.57–1.52) compared to those with any industry funding (0.42; 95% CI 0.27–0.64; *p* = 0.01). There was a significant difference in enrollment rates by the continent of the first author: Europe 0.57 (95% CI 0.44–0.75), North America 0.82 (95% CI 0.39–1.74), Australia 0.95 (95% CI 0.55–1.62), South America 1.85 (95% CI 0.81–4.22) and Asia 1.89 (95% CI 0.72–4.97; *p* = 0.01 for overall comparison). Trials with the first author from Asia (47%) and South America (38%) were more likely to be single center trials than those originating from North America (19%) and Europe (13%) (Additional file 1: Appendix E).

Enrollment rates did not differ by publication year (2009–2013: 0.62; 95% CI 0.41–0.92 vs 2014–2019: 1.08; 95% CI 0.63–1.85; *p* = 0.10), intervention (pharmaceutical: 0.81; 95% CI 0.44–1.50 vs non-pharmaceutical: 0.85; 95% CI 0.56–1.31; *p* = 0.90), or method of informed consent (retrospective permitted: 0.65; 95% CI 0.48–0.89 vs prospective required: 0.89; 95% CI 0.59–1.35; *p* = 0.25). Detailed article-level and summary results by study characteristics are available in Additional file 1: Appendices F through P.

### Achievement of target enrollment

Four trials did not report a target sample size. Of the 90 trials reporting a target sample size, 36 trials (40%) failed to enroll at least 95% of the target sample size. The most commonly cited reasons for trials falling short of their enrollment target included intervention futility (*n* = 15; 42%), safety concern (*n* = 10; 28%), and slow enrollment (*n* = 8; 22%). Characteristics of trials that cited slow enrollment for falling short of their enrollment target are included in Additional file 1: Appendix Q. Enrollment rates did not differ between trials that failed to reach 95% of the targeted sample size (0.59; 95% CI 0.38–0.91) compared to trials that met or exceeded 95% of the targeted sample size (1.03; 95% CI 0.62–1.72; *p* = 0.10).

## Discussion

In this systematic review of 94 contemporary ARDS/ALI and sepsis trials, we found an overall enrollment rate of less than 1 participant per month per site, and almost 1 in 10 trials failed to meet the target sample size due to slow enrollment. These findings have important implications for trial planning and provide evidence to support the general perception among critical care trialists that conducting trials among critically ill patients is arduous, and novel interventions to improve enrollment, trial efficiencies, and alternative designs are needed [12].

Our overall findings are consistent with a previous study that found a median enrollment rate of 0.90 patients per site per month among 23 multicenter RCTs of non-surgical interventions for adult critically ill patients [20]. Another prior review found that $\ge \hspace{0.17em}$95% of target enrollment was not achieved in 15% of adult critical care trials due to recruitment or logistical issues [6]. Although this proportion was lower in our focused review of ALI/ARDS and sepsis trials, both are likely to be underestimates since trials that fail to achieve enrollment targets may be more likely to remain unpublished or be published in lower-impact journals, which were not included in either review. Furthermore, studies that are underpowered or stopped early due to enrollment difficulties expose enrolled participants to potential risk with reduced likelihood of benefit, thus raising ethical concerns in terms of not providing societal benefit [10].

The overall enrollment rate among sepsis trials was nearly twice that in ARDS/ALI trials, which is likely reflective of important clinical differences between these syndromes that may impact research recruitment processes. For example, patients with ARDS/ALI are likely to be mechanically ventilated and therefore reliant on surrogate decision-makers for research participation decisions. Surrogates often feel overwhelmed by the acuity of the patient’s illness and substituted judgment process, which influences their decision-making processes for research participation [21]. Use of ethical and effective behavioral economic nudges during recruitment may help to minimize the surrogate decision-making process and promote informed trial participation [22, 23].

We found several trial-related factors that were associated with better enrollment rates that may help to inform future trial planning. Single-center and non-industry funded trials had higher enrollment rates, which may indicate potential participants’ greater trust and confidence in the medical and research teams, important factors in surrogate decision-makers’ research participation decisions [24]. Single-center trials may recruit more rapidly in part due to increased motivation of recruiters who likely know or may even be the principal investigator (PI) of the trial. In contrast, multi-center trials face a higher burden to provide consistent recruitment oversight and troubleshooting for site PIs who are crucial to recruitment efforts but also likely to have competing demands on their time. Additionally, there may be differences in the motivation of those recruiting based on the funding source. While adding sites may not be the most efficient solution for trials struggling with slow enrollment, this decision must be balanced with the limited generalizability of evidence from single-center trials. Geographic differences in enrollment rates may partly be explained by differences in the use of single vs multicenter trials and varying requirements for regulatory controls. Interestingly, neither the timing of consent (prospective required versus retrospective permitted), nor type of intervention was associated with enrollment rate in this sample of clinical trials.

Previous qualitative studies have identified factors important to enrollment decisions made by surrogates of critically ill patients. In addition to the importance of trust [21, 25], those who elected to enroll the patient cited that they had a desire to help future patients, were following the perceived wish of the patient, had a generally favorable view of research, and perceived the trial as safe. Those who declined enrollment cited the acuity of the patient’s illness, a fear of “rocking the boat”, and concern with potential interference with the medical team’s decisions [4]. The experiences in COVID-19 ICU trial enrollment suggest there are likely additional influential factors on ICU surrogates’ decision-making for research participation. For example, the CoDEX trial reported an enrollment rate of 3.6 participants per month per site [26], the HENIVOT trial 13.8 participants per month per site [27], and the INSPIRATION trial 15 participants per month per site [28]. These enrollment rates are significantly higher than what we found in this review of ICU trials conducted before the pandemic, and may be attributable to the allocation of increased resources for recruitment, a feeling of desperation among COVID patients and surrogates motivated to try anything for this unknown and life-threatening disease, or the high volume of COVID patients in the ICU. While research conducted during the COVID-19 pandemic represents an extreme example that is not likely to generalize to most other critical care trials, there may be lessons to glean nonetheless about other important influences on enrollment processes and decisions for participation in critical care research.

Despite multi-center trials being common in this sample, recruitment still required a median of three years to complete. Although the number of eligible patients at each site was inconsistently reported and thus was not included in our analysis, given the ubiquity of ARDS/ALI and sepsis in the ICU [29], it is unlikely that a lack of eligible participants is the primary limiting factor. Indeed, a multicenter study of research recruitment outcomes among critically ill patients found that over half of the opportunities to recruit an eligible patient failed, most commonly due to the research team being unavailable or an inability to contact a surrogate decision-maker [5]. Increasing the number of personnel involved in screening and approaching eligible participants may be an efficient way of increasing the trial enrollment rate, but the increased cost of doing so must also be considered.

Several limitations must be considered when interpreting the results. First, this report only includes trials that enrolled patients with ARDS/ALI or sepsis and were published in select journals. While this intentionally narrow scope may limit the generalizability of the results, we focused on the most common critical illness syndromes and high-impact journals as this is the evidence base with the greatest impact on clinical practice. It is likely that trials that experience very slow enrollment or other difficulties achieving their enrollment targets are more likely to be published in lower-impact journals or go unpublished. Therefore, our results likely represent an overestimation of enrollment rates and an underestimation of the problem. Second, due to a lack of reporting of site-level recruitment metrics, our analysis assumes that each site was actively enrolling throughout the duration of the trial. This assumption is likely to be inaccurate for some studies and may have led to an underestimate in the overall point estimates in this review. Third, site- and provider-level factors could play an important role in enrollment, however, important details about such factors are rarely reported in publications. Future research focused on collecting and analyzing site- and provider-level factors may improve our understanding of factors contributing to successful trial enrollment. Fourth, due to the small numbers in some subgroups, our subgroup analyses may be underpowered. Lastly, we could not determine the overall proportion of eligible participants approached who were enrolled (consent rate) due to significant heterogeneity in reporting. Indeed, adherence to CONSORT reporting guidelines has been notably poor among critical care trials [30], which often experience unique recruitment circumstances (e.g., clinicians declining participation on behalf of the patient or reliance on surrogate decision-makers). Adoption of a standard CONSORT diagram is needed to facilitate an accurate evaluation of consent rates.

## Conclusion

Our review of 94 recent high-impact ARDS/ALI and sepsis trials revealed that enrollment challenges remain an important source of delay in new evidence generation and threaten the scientific validity of trial findings. Novel strategies that surmount modifiable enrollment barriers are urgently needed to improve the efficiency of critical care trials to enable rapid implementation of evidence-based interventions into clinical practice.

## Supplementary Information

**Additional file 1.** Supplemental information and data. Includes the PRISMA checklist, complete online search strategy, citations for articles included in systematic review and subgroup analyses.

## Data Availability

The datasets used during the current study are available from the corresponding author on reasonable request.
