# Supplementary material for: A systematic review and meta-analysis of enrollment into ARDS and sepsis trials published between 2009 and 2019 in major journals
Source: Crit Care. 2021 Nov 15;25:392. doi: 10.1186/s13054-021-03804-1 (PMC8591428; doi:10.1186/s13054-021-03804-1)
Supplement: Supplementary file 1 — Additional file 1. Supplemental information and data. Includes the PRISMA checklist, complete online search strategy, citations for articles included in systematic review and subgroup analyses. [file 13054_2021_3804_MOESM1_ESM.docx]

**Online Supplement**

| Appendix | Description | Type | Page |
| --- | --- | --- | --- |
| A | PRISMA 2020 Checklist | Table | 2 |
| B | Complete online search strategy | Table | 4 |
| C | Citations for articles included in systematic review (n=94) | Table | 5 |
| D | Characteristics of patient enrollment | Table | 9 |
| E | Single center vs multicenter by continent | Table | 10 |
| F | Article-level and summary results for monthly enrollment per site | Figure | 11 |
| G | Article-level and summary results for monthly enrollment per site for sepsis trials only | Figure | 13 |
| H | Article-level and summary results for monthly enrollment per site for ARDS trials only | Figure | 15 |
| I | Article-level and summary results for monthly enrollment per site by patient population (Sepsis vs. ARDS) | Figure | 16 |
| J | Article-level and summary results for monthly enrollment per site by time period (2009-2014 vs. 2015-2019) | Figure | 18 |
| K | Article-level and summary results for monthly enrollment per site by funding sources (industry vs. non- industry) | Figure | 20 |
| L | Article-level and summary results for monthly enrollment per site by intervention (drug vs. non-drug) | Figure | 22 |
| M | Article-level and summary results for monthly enrollment per site by continent | Figure | 24 |
| N | Article-level and summary results for monthly enrollment per site by consent type (prospective vs waived) | Figure | 26 |
| O | Article-level and summary results for monthly enrollment per site by enrollment target achieved | Figure | 28 |
| P | Article-level and summary results for monthly enrollment per site by single center vs multicenter | Figure | 30 |
| Q | Characteristics of studies which stopped early citing enrollment difficulties (n=8) | Table | 32 |

Appendix A: PRISMA 2020 checklist

| **Section and Topic** | **Item #** | **Checklist item** | **Location where item is reported** |
| --- | --- | --- | --- |
| **TITLE** | | |  |
| Title | 1 | Identify the report as a systematic review. | Title |
| **ABSTRACT** | | |  |
| Abstract | 2 | See the PRISMA 2020 for Abstracts checklist. | Abstract |
| **INTRODUCTION** | | |  |
| Rationale | 3 | Describe the rationale for the review in the context of existing knowledge. | Page 6 |
| Objectives | 4 | Provide an explicit statement of the objective(s) or question(s) the review addresses. | Page 6 |
| **METHODS** | | |  |
| Eligibility criteria | 5 | Specify the inclusion and exclusion criteria for the review and how studies were grouped for the syntheses. | Page 8 |
| Information sources | 6 | Specify all databases, registers, websites, organisations, reference lists and other sources searched or consulted to identify studies. Specify the date when each source was last searched or consulted. | Page 7 |
| Search strategy | 7 | Present the full search strategies for all databases, registers and websites, including any filters and limits used. | Appendix B |
| Selection process | 8 | Specify the methods used to decide whether a study met the inclusion criteria of the review, including how many reviewers screened each record and each report retrieved, whether they worked independently, and if applicable, details of automation tools used in the process. | Page 8 |
| Data collection process | 9 | Specify the methods used to collect data from reports, including how many reviewers collected data from each report, whether they worked independently, any processes for obtaining or confirming data from study investigators, and if applicable, details of automation tools used in the process. | Page 8 |
| Data items | 10a | List and define all outcomes for which data were sought. Specify whether all results that were compatible with each outcome domain in each study were sought (e.g. for all measures, time points, analyses), and if not, the methods used to decide which results to collect. | Table 1 |
|  | 10b | List and define all other variables for which data were sought (e.g. participant and intervention characteristics, funding sources). Describe any assumptions made about any missing or unclear information. | Table 1 |
| Study risk of bias assessment | 11 | Specify the methods used to assess risk of bias in the included studies, including details of the tool(s) used, how many reviewers assessed each study and whether they worked independently, and if applicable, details of automation tools used in the process. | Page 8 |
| Effect measures | 12 | Specify for each outcome the effect measure(s) (e.g. risk ratio, mean difference) used in the synthesis or presentation of results. | Page 8 |
| Synthesis methods | 13a | Describe the processes used to decide which studies were eligible for each synthesis (e.g. tabulating the study intervention characteristics and comparing against the planned groups for each synthesis (item #5)). | Page 8 |
|  | 13b | Describe any methods required to prepare the data for presentation or synthesis, such as handling of missing summary statistics, or data conversions. | Page 8 |
|  | 13c | Describe any methods used to tabulate or visually display results of individual studies and syntheses. | Page 8 |
|  | 13d | Describe any methods used to synthesize results and provide a rationale for the choice(s). If meta-analysis was performed, describe the model(s), method(s) to identify the presence and extent of statistical heterogeneity, and software package(s) used. | Page 9 |
|  | 13e | Describe any methods used to explore possible causes of heterogeneity among study results (e.g. subgroup analysis, meta-regression). | Page 9 |
|  | 13f | Describe any sensitivity analyses conducted to assess robustness of the synthesized results. | N/A |
| Reporting bias assessment | 14 | Describe any methods used to assess risk of bias due to missing results in a synthesis (arising from reporting biases). | Figure 3 |
| Certainty assessment | 15 | Describe any methods used to assess certainty (or confidence) in the body of evidence for an outcome. | Page 9 |
| **RESULTS** | | |  |
| Study selection | 16a | Describe the results of the search and selection process, from the number of records identified in the search to the number of studies included in the review, ideally using a flow diagram. | Figure 1 |
|  | 16b | Cite studies that might appear to meet the inclusion criteria, but which were excluded, and explain why they were excluded. | Appendix P |
| Study characteristics | 17 | Cite each included study and present its characteristics. | Appendix P |
| Risk of bias in studies | 18 | Present assessments of risk of bias for each included study. | N/A |
| Results of individual studies | 19 | For all outcomes, present, for each study: (a) summary statistics for each group (where appropriate) and (b) an effect estimate and its precision (e.g. confidence/credible interval), ideally using structured tables or plots. | Figure 2 |
| Results of syntheses | 20a | For each synthesis, briefly summarise the characteristics and risk of bias among contributing studies. | N/A |
|  | 20b | Present results of all statistical syntheses conducted. If meta-analysis was done, present for each the summary estimate and its precision (e.g. confidence/credible interval) and measures of statistical heterogeneity. If comparing groups, describe the direction of the effect. | Page 10-11 |
|  | 20c | Present results of all investigations of possible causes of heterogeneity among study results. | N/A |
|  | 20d | Present results of all sensitivity analyses conducted to assess the robustness of the synthesized results. | N/A |
| Reporting biases | 21 | Present assessments of risk of bias due to missing results (arising from reporting biases) for each synthesis assessed. | N/A |
| Certainty of evidence | 22 | Present assessments of certainty (or confidence) in the body of evidence for each outcome assessed. | Page 10-11 |
| **DISCUSSION** | | |  |
| Discussion | 23a | Provide a general interpretation of the results in the context of other evidence. | Page 11 |
|  | 23b | Discuss any limitations of the evidence included in the review. | Page 14-15 |
|  | 23c | Discuss any limitations of the review processes used. | Page 14-15 |
|  | 23d | Discuss implications of the results for practice, policy, and future research. | Page 15 |
| **OTHER INFORMATION** | | |  |
| Registration and protocol | 24a | Provide registration information for the review, including register name and registration number, or state that the review was not registered. | Page 7 |
|  | 24b | Indicate where the review protocol can be accessed, or state that a protocol was not prepared. | Page 7 |
|  | 24c | Describe and explain any amendments to information provided at registration or in the protocol. | N/A |
| Support | 25 | Describe sources of financial or non-financial support for the review, and the role of the funders or sponsors in the review. | Page 15-16 |
| Competing interests | 26 | Declare any competing interests of review authors. | Page 15 |
| Availability of data, code and other materials | 27 | Report which of the following are publicly available and where they can be found: template data collection forms; data extracted from included studies; data used for all analyses; analytic code; any other materials used in the review. | Page 15 |

*From:*  Page MJ, McKenzie JE, Bossuyt PM, Boutron I, Hoffmann TC, Mulrow CD, et al. The PRISMA 2020 statement: an updated guideline for reporting systematic reviews. BMJ 2021;372:n71. doi: 10.1136/bmj.n71

For more information, visit: <http://www.prisma-statement.org/>

Appendix B: Complete online search strategy

| **Step** | **Source** | **Search Strategy** |
| --- | --- | --- |
| **1** | **2018 Journal Impact Factor**  **without Journal Self Cites**  Journal Citation Reports  (Clarivate Analytics, 2019) | **General Medicine:** The New England Journal of Medicine (70.0), Lancet (57.8), JAMA (50.1), Nature Reviews. Disease Primers (32.1), British Medical Journal (25.9), JAMA Internal Medicine (20.0), Annals of Internal Medicine (18.4)  **Critical Care:** Lancet Respiratory Medicine (22.3), Intensive Care Medicine (16.7), American Journal of Respiratory and Critical Care Medicine (14.8), Chest (9.2), Critical Care (6.6), Critical Care Medicine (6.3), Annals of the American Thoracic Society (3.7) |
| **2** | **PubMed**  1/15/2020  457  articles | (("2009"[PDAT] : "2019"[PDAT]) AND ("clinical trial"[Publication Type]) AND ((("respiratory distress syndrome, adult"[MeSH Terms]) OR "sepsis"[MeSH Terms]) OR "acute lung injury"[MeSH Terms]) AND (((((((((((((("The New England journal of medicine"[Journal]) OR "JAMA"[Journal]) OR "JAMA internal medicine"[Journal]) OR "British medical journal"[Journal]) OR "Lancet (London, England)"[Journal]) OR "Annals of internal medicine"[Journal]) OR "Chest"[Journal]) OR "Critical care medicine"[Journal]) OR "Critical care (London, England)"[Journal]) OR "Intensive care medicine"[Journal]) OR "American journal of respiratory and critical care medicine"[Journal]) OR "The Lancet. Respiratory medicine"[Journal]) OR "Annals of the American Thoracic Society"[Journal]) OR “Nature reviews. Disease primers” [Journal])) |
| **3** | **Journal Websites** | Hand search of all issues of each journal published in 2017. |

Appendix C: Citations for articles include in systematic review (n=94) Characteristics of patient enrollment

| ID | Article | Citation |
| --- | --- | --- |
| 1 | Abdul-Aziz et al. (2016) | Abdul-Aziz MH, Sulaiman H, Mat-Nor MB, et al. Beta-Lactam Infusion in Severe Sepsis (BLISS): a prospective, two-centre, open-labelled randomised controlled trial of continuous versus intermittent beta-lactam infusion in critically ill patients with severe sepsis. Intensive Care Med. 2016;42(10):1535-1545. |
| 2 | Andrews et al. (2014) | Andrews B, Muchemwa L, Kelly P, Lakhi S, Heimburger DC, Bernard GR. Simplified severe sepsis protocol: a randomized controlled trial of modified early goal-directed therapy in Zambia. Crit Care Med. 2014;42(11):2315-2324. |
| 3 | Andrews et al. (2017) | Andrews B, Semler MW, Muchemwa L, et al. Effect of an Early Resuscitation Protocol on In-hospital Mortality Among Adults With Sepsis and Hypotension: A Randomized Clinical Trial. Jama. 2017;318(13):1233-1240. |
| 4 | Angurana et al. (2018) | Angurana SK, Bansal A, Singhi S, et al. Evaluation of Effect of Probiotics on Cytokine Levels in Critically Ill Children With Severe Sepsis: A Double-Blind, Placebo-Controlled Trial. Crit Care Med. 2018;46(10):1656-1664. |
| 5 | Annane et al. (2010) | Annane D, Cariou A, Maxime V, et al. Corticosteroid treatment and intensive insulin therapy for septic shock in adults: a randomized controlled trial. Jama. 2010;303(4):341-348. |
| 6 | Annane et al. (2013) | Annane D, Timsit JF, Megarbane B, et al. Recombinant human activated protein C for adults with septic shock: a randomized controlled trial. Am J Respir Crit Care Med. 2013;187(10):1091-1097. |
| 7 | Annane et al. (2018) | Annane D, Renault A, Brun-Buisson C, et al. Hydrocortisone plus Fludrocortisone for Adults with Septic Shock. N Engl J Med. 2018;378(9):809-818. |
| 8 | Asfar et al. (2014) | Asfar P, Meziani F, Hamel JF, et al. High versus low blood-pressure target in patients with septic shock. N Engl J Med. 2014;370(17):1583-1593. |
| 9 | Asfar et al. (2017) | Asfar P, Schortgen F, Boisrame-Helms J, et al. Hyperoxia and hypertonic saline in patients with septic shock (HYPERS2S): a two-by-two factorial, multicentre, randomised, clinical trial. Lancet Respir Med. 2017;5(3):180-190. |
| 10 | Barbar et al. (2018) | Barbar SD, Clere-Jehl R, Bourredjem A, et al. Timing of Renal-Replacement Therapy in Patients with Acute Kidney Injury and Sepsis. N Engl J Med. 2018;379(15):1431-1442. |
| 11 | Barbosa et al. (2010) | Barbosa VM, Miles EA, Calhau C, Lafuente E, Calder PC. Effects of a fish oil containing lipid emulsion on plasma phospholipid fatty acids, inflammatory markers, and clinical outcomes in septic patients: a randomized, controlled clinical trial. Crit Care. 2010;14(1):R5. |
| 12 | Bein et al. (2013) | Bein T, Weber-Carstens S, Goldmann A, et al. Lower tidal volume strategy ( approximately 3 ml/kg) combined with extracorporeal CO2 removal versus 'conventional' protective ventilation (6 ml/kg) in severe ARDS: the prospective randomized Xtravent-study. Intensive Care Med. 2013;39(5):847-856. |
| 13 | Bergamin et al. (2017) | Bergamin FS, Almeida JP, Landoni G, et al. Liberal Versus Restrictive Transfusion Strategy in Critically Ill Oncologic Patients: The Transfusion Requirements in Critically Ill Oncologic Patients Randomized Controlled Trial. Crit Care Med. 2017;45(5):766-773. |
| 14 | Bloos et al. (2016) | Bloos F, Trips E, Nierhaus A, et al. Effect of Sodium Selenite Administration and Procalcitonin-Guided Therapy on Mortality in Patients With Severe Sepsis or Septic Shock: A Randomized Clinical Trial. JAMA Intern Med. 2016;176(9):1266-1276. |
| 15 | Boerma et al. (2010) | Boerma EC, Koopmans M, Konijn A, et al. Effects of nitroglycerin on sublingual microcirculatory blood flow in patients with severe sepsis/septic shock after a strict resuscitation protocol: a double-blind randomized placebo controlled trial. Crit Care Med. 2010;38(1):93-100. |
| 16 | Brocklehurst et al. (2011) | Brocklehurst P, Farrell B, King A, et al. Treatment of neonatal sepsis with intravenous immune globulin. N Engl J Med. 2011;365(13):1201-1211. |
| 17 | Brunkhorst et al. (2012) | Brunkhorst FM, Oppert M, Marx G, et al. Effect of empirical treatment with moxifloxacin and meropenem vs meropenem on sepsis-related organ dysfunction in patients with severe sepsis: a randomized trial. Jama. 2012;307(22):2390-2399. |
| 18 | Caironi et al. (2014) | Caironi P, Tognoni G, Masson S, et al. Albumin replacement in patients with severe sepsis or septic shock. N Engl J Med. 2014;370(15):1412-1421. |
| 19 | Cavalcanti et al. (2017) | Cavalcanti AB, Suzumura EA, Laranjeira LN, et al. Effect of Lung Recruitment and Titrated Positive End-Expiratory Pressure (PEEP) vs Low PEEP on Mortality in Patients With Acute Respiratory Distress Syndrome: A Randomized Clinical Trial. Jama. 2017;318(14):1335-1345. |
| 20 | Chung et al. (2017) | Chung KK, Coates EC, Smith DJ, Jr., et al. High-volume hemofiltration in adult burn patients with septic shock and acute kidney injury: a multicenter randomized controlled trial. Crit Care. 2017;21(1):289. |
| 21 | Combes et al. (2018) | Combes A, Hajage D, Capellier G, et al. Extracorporeal Membrane Oxygenation for Severe Acute Respiratory Distress Syndrome. N Engl J Med. 2018;378(21):1965-1975. |
| 22 | Craig et al. (2011) | Craig TR, Duffy MJ, Shyamsundar M, et al. A randomized clinical trial of hydroxymethylglutaryl- coenzyme a reductase inhibition for acute lung injury (The HARP Study). Am J Respir Crit Care Med. 2011;183(5):620-626. |
| 23 | Cruz et al. (2009) | Cruz DN, Antonelli M, Fumagalli R, et al. Early use of polymyxin B hemoperfusion in abdominal septic shock: the EUPHAS randomized controlled trial. Jama. 2009;301(23):2445-2452. |
| 24 | Dellinger et al. (2018) | Dellinger RP, Bagshaw SM, Antonelli M, et al. Effect of Targeted Polymyxin B Hemoperfusion on 28-Day Mortality in Patients With Septic Shock and Elevated Endotoxin Level: The EUPHRATES Randomized Clinical Trial. Jama. 2018;320(14):1455-1463. |
| 25 | Dhainaut et al. (2009) | Dhainaut JF, Antonelli M, Wright P, et al. Extended drotrecogin alfa (activated) treatment in patients with prolonged septic shock. Intensive Care Med. 2009;35(7):1187-1195. |
| 26 | Dulhunty et al. (2015) | Dulhunty JM, Roberts JA, Davis JS, et al. A Multicenter Randomized Trial of Continuous versus Intermittent beta-Lactam Infusion in Severe Sepsis. Am J Respir Crit Care Med. 2015;192(11):1298-1305. |
| 27 | Ferguson et al. (2013) | Ferguson ND, Cook DJ, Guyatt GH, et al. High-frequency oscillation in early acute respiratory distress syndrome. N Engl J Med. 2013;368(9):795-805. |
| 28 | Fowler et al. (2019) | Fowler AA, 3rd, Truwit JD, Hite RD, et al. Effect of Vitamin C Infusion on Organ Failure and Biomarkers of Inflammation and Vascular Injury in Patients With Sepsis and Severe Acute Respiratory Failure: The CITRIS-ALI Randomized Clinical Trial. Jama. 2019;322(13):1261-1270. |
| 29 | Gando et al. (2013) | Gando S, Saitoh D, Ishikura H, et al. A randomized, controlled, multicenter trial of the effects of antithrombin on disseminated intravascular coagulation in patients with sepsis. Crit Care. 2013;17(6):R297. |
| 30 | Gao Smith et al. (2012) | Gao Smith F, Perkins GD, Gates S, et al. Effect of intravenous beta-2 agonist treatment on clinical outcomes in acute respiratory distress syndrome (BALTI-2): a multicentre, randomised controlled trial. Lancet. 2012;379(9812):229-235. |
| 31 | Gordon et al. (2016) | Gordon AC, Mason AJ, Thirunavukkarasu N, et al. Effect of Early Vasopressin vs Norepinephrine on Kidney Failure in Patients With Septic Shock: The VANISH Randomized Clinical Trial. Jama. 2016;316(5):509-518. |
| 32 | Guerin et al. (2013) | Guerin C, Reignier J, Richard JC, et al. Prone positioning in severe acute respiratory distress syndrome. N Engl J Med. 2013;368(23):2159-2168. |
| 33 | Guidet et al. (2012) | Guidet B, Martinet O, Boulain T, et al. Assessment of hemodynamic efficacy and safety of 6% hydroxyethylstarch 130/0.4 vs. 0.9% NaCl fluid replacement in patients with severe sepsis: the CRYSTMAS study. Crit Care. 2012;16(3):R94. |
| 34 | Hernandez et al. (2019) | Hernandez G, Ospina-Tascon GA, Damiani LP, et al. Effect of a Resuscitation Strategy Targeting Peripheral Perfusion Status vs Serum Lactate Levels on 28-Day Mortality Among Patients With Septic Shock: The ANDROMEDA-SHOCK Randomized Clinical Trial. Jama. 2019;321(7):654-664. |
| 35 | Holst et al. (2014) | Holst LB, Haase N, Wetterslev J, et al. Lower versus higher hemoglobin threshold for transfusion in septic shock. N Engl J Med. 2014;371(15):1381-1391. |
| 36 | Huh et al. (2009) | Huh JW, Jung H, Choi HS, Hong SB, Lim CM, Koh Y. Efficacy of positive end-expiratory pressure titration after the alveolar recruitment manoeuvre in patients with acute respiratory distress syndrome. Crit Care. 2009;13(1):R22. |
| 37 | Igonin et al. (2012) | Igonin AA, Protsenko DN, Galstyan GM, et al. C1-esterase inhibitor infusion increases survival rates for patients with sepsis*. Crit Care Med. 2012;40(3):770-777. |
| 38 | Itenov et al. (2018) | Itenov TS, Johansen ME, Bestle M, et al. Induced hypothermia in patients with septic shock and respiratory failure (CASS): a randomised, controlled, open-label trial. Lancet Respir Med. 2018;6(3):183-192. |
| 39 | Jaimes et al. (2009) | Jaimes F, De La Rosa G, Morales C, et al. Unfractioned heparin for treatment of sepsis: A randomized clinical trial (The HETRASE Study). Crit Care Med. 2009;37(4):1185-1196. |
| 40 | Jeon et al. (2018) | Jeon K, Song JU, Chung CR, Yang JH, Suh GY. Incidence of hypotension according to the discontinuation order of vasopressors in the management of septic shock: a prospective randomized trial (DOVSS). Crit Care. 2018;22(1):131. |
| 41 | Joannes-Boyau et al. (2013) | Joannes-Boyau O, Honore PM, Perez P, et al. High-volume versus standard-volume haemofiltration for septic shock patients with acute kidney injury (IVOIRE study): a multicentre randomized controlled trial. Intensive Care Med. 2013;39(9):1535-1546. |
| 42 | Jones et al. (2010) | Jones AE, Shapiro NI, Trzeciak S, Arnold RC, Claremont HA, Kline JA. Lactate clearance vs central venous oxygen saturation as goals of early sepsis therapy: a randomized clinical trial. Jama. 2010;303(8):739-746. |
| 43 | Kawazoe et al. (2017) | Kawazoe Y, Miyamoto K, Morimoto T, et al. Effect of Dexmedetomidine on Mortality and Ventilator-Free Days in Patients Requiring Mechanical Ventilation With Sepsis: A Randomized Clinical Trial. Jama. 2017;317(13):1321-1328. |
| 44 | Keh et al. (2016) | Keh D, Trips E, Marx G, et al. Effect of Hydrocortisone on Development of Shock Among Patients With Severe Sepsis: The HYPRESS Randomized Clinical Trial. Jama. 2016;316(17):1775-1785. |
| 45 | Kesecioglu et al. (2009) | Kesecioglu J, Beale R, Stewart TE, et al. Exogenous natural surfactant for treatment of acute lung injury and the acute respiratory distress syndrome. Am J Respir Crit Care Med. 2009;180(10):989-994. |
| 46 | Lalgudi Ganesan et al. (2018) | Lalgudi Ganesan S, Jayashree M, Chandra Singhi S, Bansal A. Airway Pressure Release Ventilation in Pediatric Acute Respiratory Distress Syndrome. A Randomized Controlled Trial. Am J Respir Crit Care Med. 2018;198(9):1199-1207. |
| 47 | Leone et al. (2014) | Leone M, Bechis C, Baumstarck K, et al. De-escalation versus continuation of empirical antimicrobial treatment in severe sepsis: a multicenter non-blinded randomized noninferiority trial. Intensive Care Med. 2014;40(10):1399-1408. |
| 48 | Liu et al. (2018) | Liu ZM, Chen J, Kou Q, et al. Terlipressin versus norepinephrine as infusion in patients with septic shock: a multicentre, randomised, double-blinded trial. Intensive Care Med. 2018;44(11):1816-1825. |
| 49 | Matthay et al. (2011) | Matthay MA, Brower RG, Carson S, et al. Randomized, placebo-controlled clinical trial of an aerosolized beta(2)-agonist for treatment of acute lung injury. Am J Respir Crit Care Med. 2011;184(5):561-568. |
| 50 | McAuley et al. (2014) | McAuley DF, Laffey JG, O'Kane CM, et al. Simvastatin in the acute respiratory distress syndrome. N Engl J Med. 2014;371(18):1695-1703. |
| 51 | Meisel et al. (2009) | Meisel C, Schefold JC, Pschowski R, et al. Granulocyte-macrophage colony-stimulating factor to reverse sepsis-associated immunosuppression: a double-blind, randomized, placebo-controlled multicenter trial. Am J Respir Crit Care Med. 2009;180(7):640-648. |
| 52 | Moss et al. (2019) | Moss M, Huang DT, Brower RG, et al. Early Neuromuscular Blockade in the Acute Respiratory Distress Syndrome. N Engl J Med. 2019;380(21):1997-2008. |
| 53 | Mouncey et al. (2015) | Mouncey PR, Osborn TM, Power GS, et al. Trial of early, goal-directed resuscitation for septic shock. N Engl J Med. 2015;372(14):1301-1311. |
| 54 | Oliveira et al. (2013) | Oliveira CF, Botoni FA, Oliveira CR, et al. Procalcitonin versus C-reactive protein for guiding antibiotic therapy in sepsis: a randomized trial. Crit Care Med. 2013;41(10):2336-2343. |
| 55 | Opal et al. (2013) | Opal SM, Laterre PF, Francois B, et al. Effect of eritoran, an antagonist of MD2-TLR4, on mortality in patients with severe sepsis: the ACCESS randomized trial. Jama. 2013;309(11):1154-1162. |
| 56 | Paine et al. (2012) | Paine R, 3rd, Standiford TJ, Dechert RE, et al. A randomized trial of recombinant human granulocyte-macrophage colony stimulating factor for patients with acute lung injury. Crit Care Med. 2012;40(1):90-97. |
| 57 | Palizas et al. (2009) | Palizas F, Dubin A, Regueira T, et al. Gastric tonometry versus cardiac index as resuscitation goals in septic shock: a multicenter, randomized, controlled trial. Crit Care. 2009;13(2):R44. |
| 58 | Papazian et al. (2010) | Papazian L, Forel JM, Gacouin A, et al. Neuromuscular blockers in early acute respiratory distress syndrome. N Engl J Med. 2010;363(12):1107-1116. |
| 59 | Pappalardo et al. (2016) | Pappalardo F, Crivellari M, Di Prima AL, et al. Protein C zymogen in severe sepsis: a double-blinded, placebo-controlled, randomized study. Intensive Care Med. 2016;42(11):1706-1714. |
| 60 | Patel et al. (2016) | Patel BK, Wolfe KS, Pohlman AS, Hall JB, Kress JP. Effect of Noninvasive Ventilation Delivered by Helmet vs Face Mask on the Rate of Endotracheal Intubation in Patients With Acute Respiratory Distress Syndrome: A Randomized Clinical Trial. Jama. 2016;315(22):2435-2441. |
| 61 | Payen et al. (2009) | Payen DM, Guilhot J, Launey Y, et al. Early use of polymyxin B hemoperfusion in patients with septic shock due to peritonitis: a multicenter randomized control trial. Intensive Care Med. 2015;41(6):975-984. |
| 62 | Payen et al. (2015) | Payen D, Mateo J, Cavaillon JM, Fraisse F, Floriot C, Vicaut E. Impact of continuous venovenous hemofiltration on organ failure during the early phase of severe sepsis: a randomized controlled trial. Crit Care Med. 2009;37(3):803-810. |
| 63 | Peake et al. (2014) | Peake SL, Delaney A, Bailey M, et al. Goal-directed resuscitation for patients with early septic shock. N Engl J Med. 2014;371(16):1496-1506. |
| 64 | Perner et al. (2012) | Perner A, Haase N, Guttormsen AB, et al. Hydroxyethyl starch 130/0.42 versus Ringer's acetate in severe sepsis. N Engl J Med. 2012;367(2):124-134. |
| 65 | Pontes-Arruda et al. (2011) | Pontes-Arruda A, Martins LF, de Lima SM, et al. Enteral nutrition with eicosapentaenoic acid, gamma-linolenic acid and antioxidants in the early treatment of sepsis: results from a multicenter, prospective, randomized, double-blinded, controlled study: the INTERSEPT study. Crit Care. 2011;15(3):R144. |
| 66 | Quenot et al. (2015) | Quenot JP, Binquet C, Vinsonneau C, et al. Very high volume hemofiltration with the Cascade system in septic shock patients. Intensive Care Med. 2015;41(12):2111-2120. |
| 67 | Quraishi et al. (2015) | Quraishi SA, De Pascale G, Needleman JS, et al. Effect of Cholecalciferol Supplementation on Vitamin D Status and Cathelicidin Levels in Sepsis: A Randomized, Placebo-Controlled Trial. Crit Care Med. 2015;43(9):1928-1937. |
| 68 | Ranieri et al. (2012) | Ranieri VM, Thompson BT, Barie PS, et al. Drotrecogin alfa (activated) in adults with septic shock. N Engl J Med. 2012;366(22):2055-2064. |
| 69 | Rice et al. (2010) | Rice TW, Wheeler AP, Bernard GR, et al. A randomized, double-blind, placebo-controlled trial of TAK-242 for the treatment of severe sepsis. Crit Care Med. 2010;38(8):1685-1694. |
| 70 | Rice et al. (2011) | Rice TW, Wheeler AP, Thompson BT, deBoisblanc BP, Steingrub J, Rock P. Enteral omega-3 fatty acid, gamma-linolenic acid, and antioxidant supplementation in acute lung injury. Jama. 2011;306(14):1574-1581. |
| 71 | Rice et al. (2012) | Rice TW, Wheeler AP, Thompson BT, et al. Initial trophic vs full enteral feeding in patients with acute lung injury: the EDEN randomized trial. Jama. 2012;307(8):795-803. |
| 72 | Richard et al. (2015) | Richard JC, Bayle F, Bourdin G, et al. Preload dependence indices to titrate volume expansion during septic shock: a randomized controlled trial. Crit Care. 2015;19:5. |
| 73 | Savioli et al. (2009) | Savioli M, Cugno M, Polli F, et al. Tight glycemic control may favor fibrinolysis in patients with sepsis. Crit Care Med. 2009;37(2):424-431. |
| 74 | Shehabi et al. (2014) | Shehabi Y, Sterba M, Garrett PM, et al. Procalcitonin algorithm in critically ill adults with undifferentiated infection or suspected sepsis. A randomized controlled trial. Am J Respir Crit Care Med. 2014;190(10):1102-1110. |
| 75 | Shimizu et al. (2018) | Shimizu K, Yamada T, Ogura H, et al. Synbiotics modulate gut microbiota and reduce enteritis and ventilator-associated pneumonia in patients with sepsis: a randomized controlled trial. Crit Care. 2018;22(1):239. |
| 76 | Spragg et al. (2011) | Spragg RG, Taut FJ, Lewis JF, et al. Recombinant surfactant protein C-based surfactant for patients with severe direct lung injury. Am J Respir Crit Care Med. 2011;183(8):1055-1061. |
| 77 | Srisawat et al. (2018) | Srisawat N, Tungsanga S, Lumlertgul N, et al. The effect of polymyxin B hemoperfusion on modulation of human leukocyte antigen DR in severe sepsis patients. Crit Care. 2018;22(1):279. |
| 78 | Stocker et al. (2017) | Stocker M, van Herk W, El Helou S, et al. Procalcitonin-guided decision making for duration of antibiotic therapy in neonates with suspected early-onset sepsis: a multicentre, randomised controlled trial (NeoPIns). Lancet. 2017;390(10097):871-881. |
| 79 | Taccone et al. (2009) | Taccone P, Pesenti A, Latini R, et al. Prone positioning in patients with moderate and severe acute respiratory distress syndrome: a randomized controlled trial. Jama. 2009;302(18):1977-1984. |
| 80 | Tongyoo et al. (2016) | Tongyoo S, Permpikul C, Mongkolpun W, et al. Hydrocortisone treatment in early sepsis-associated acute respiratory distress syndrome: results of a randomized controlled trial. Crit Care. 2016;20(1):329. |
| 81 | Trof et al. (2012) | Trof RJ, Beishuizen A, Cornet AD, de Wit RJ, Girbes AR, Groeneveld AB. Volume-limited versus pressure-limited hemodynamic management in septic and nonseptic shock. Crit Care Med. 2012;40(4):1177-1185. |
| 82 | Truwit et al. (2014) | Truwit JD, Bernard GR, Steingrub J, et al. Rosuvastatin for sepsis-associated acute respiratory distress syndrome. N Engl J Med. 2014;370(23):2191-2200. |
| 83 | Valenta et al. (2011) | Valenta J, Brodska H, Drabek T, Hendl J, Kazda A. High-dose selenium substitution in sepsis: a prospective randomized clinical trial. Intensive Care Med. 2011;37(5):808-815. |
| 84 | Venkatesh et al. (2018) | Venkatesh B, Finfer S, Cohen J, et al. Adjunctive Glucocorticoid Therapy in Patients with Septic Shock. N Engl J Med. 2018;378(9):797-808. |
| 85 | Ventura et al. (2015) | Ventura AM, Shieh HH, Bousso A, et al. Double-Blind Prospective Randomized Controlled Trial of Dopamine Versus Epinephrine as First-Line Vasoactive Drugs in Pediatric Septic Shock. Crit Care Med. 2015;43(11):2292-2302. |
| 86 | Vincent et al. (2015) | Vincent JL, Marshall JC, Dellinger RP, et al. Talactoferrin in Severe Sepsis: Results From the Phase II/III Oral tAlactoferrin in Severe sepsIS Trial. Crit Care Med. 2015;43(9):1832-1838. |
| 87 | Vincent et al. (2019) | Vincent JL, Francois B, Zabolotskikh I, et al. Effect of a Recombinant Human Soluble Thrombomodulin on Mortality in Patients With Sepsis-Associated Coagulopathy: The SCARLET Randomized Clinical Trial. Jama. 2019;321(20):1993-2002. |
| 88 | Willson et al. (2015) | Willson DF, Truwit JD, Conaway MR, Traul CS, Egan EE. The Adult Calfactant in Acute Respiratory Distress Syndrome Trial. Chest. 2015;148(2):356-364. |
| 89 | Wu et al. (2013) | Wu J, Zhou L, Liu J, et al. The efficacy of thymosin alpha 1 for severe sepsis (ETASS): a multicenter, single-blind, randomized and controlled trial. Crit Care. 2013;17(1):R8. |
| 90 | Yealy et al. (2014) | Yealy DM, Kellum JA, Huang DT, et al. A randomized trial of protocol-based care for early septic shock. N Engl J Med. 2014;370(18):1683-1693. |
| 91 | Young et al. (2013) | Young D, Lamb SE, Shah S, et al. High-frequency oscillation for acute respiratory distress syndrome. N Engl J Med. 2013;368(9):806-813. |
| 92 | Zhan et al. (2012) | Zhan Q, Sun B, Liang L, et al. Early use of noninvasive positive pressure ventilation for acute lung injury: a multicenter randomized controlled trial. Crit Care Med. 2012;40(2):455-460. |
| 93 | Zhang et al. (2015) | Zhang Z, Ni H, Qian Z. Effectiveness of treatment based on PiCCO parameters in critically ill patients with septic shock and/or acute respiratory distress syndrome: a randomized controlled trial. Intensive Care Med. 2015;41(3):444-451. |
| 94 | Zhou et al. (2017) | Zhou X, Liu D, Su L, et al. Use of stepwise lactate kinetics-oriented hemodynamic therapy could improve the clinical outcomes of patients with sepsis-associated hyperlactatemia. Crit Care. 2017;21(1):33. |

Appendix D: Characteristics of patient enrollment

| **Characteristic** | **Number of Articles** | **Mean (SD)** | **Median [IQR]** |
| --- | --- | --- | --- |
|  |  |  |  |
| Patient enrollment | 94 | 509.9 (648.0) | 314.0 [112.8, 587.0] |
| Patient enrollment target | 90 | 652.4 (708.5) | 390.0 [150.5, 1000.0] |
| Percentage of patient enrollment target achieved | 90 | 94.1 (104.7) | 100.0 [56.2, 101.0] |
|  |  |  |  |
| Months of enrollment | 92 | 36.7 (17.5) | 36.0 [24.0, 48.2] |
| Patient enrollment per month | 92 | 14.8 (15.7) | 11.6 [3.1, 20.7] |
| Patient enrollment target per month | 88 | 18.8 (19.2) | 11.8 [4.9, 26.6] |
|  |  |  |  |
| Number of sites | 94 | 31.7 (49.4) | 18.0 [2.2, 37.8] |
| Patient enrollment per site | 94 | 56.2 (119.2) | 22.9 [11.4, 51.1] |
| Patient enrollment target per site | 90 | 58.8 (79.0) | 29.9 [15.1, 57.5] |
| Patient enrollment per site per month | 92 | 3.0 (10.1) | 0.8 [0.4, 1.7] |

Appendix E. Single center vs multicenter by continent

| **Continent** | **Single Center (%)** | **Multicenter [%]** |
| --- | --- | --- |
| Asia | 7 (47) | 8 (53) |
| Australia | 0 | 4 (100) |
| Europe | 6 (13) | 40 (87) |
| North America | 4 (19) | 17 (81) |
| South America | 3 (38) | 5 (63) |

Appendix F: Individual article and summary results for monthly enrollment per site

Number of studies combined: k = 92

rate 95%-CI

Fixed effect model 5.8305 [5.6752; 5.9900]

Random effects model 0.8306 [0.5687; 1.2130]

Quantifying heterogeneity:

tau^2 = 3.3690 [1.9076; 3.9063]; tau = 1.8355 [1.3812; 1.9764];

I^2 = 99.5% [99.4%; 99.5%]; H = 13.59 [13.19; 14.01]

Test of heterogeneity:

Q d.f. p-value

16818.05 91 0

Details on meta-analytical method:

- Inverse variance method

- DerSimonian-Laird estimator for tau^2

- Jackson method for confidence interval of tau^2 and tau

- Log transformation

Appendix G. Article-level and summary results for monthly enrollment per for sepsis trials only

Number of studies combined: k = 65

rate 95%-CI

Fixed effect model 7.5021 [7.2819; 7.7289]

Random effects model 0.9830 [0.6193; 1.5601]

Quantifying heterogeneity:

tau^2 = 3.5514 [1.9664; 4.6157]; tau = 1.8845 [1.4023; 2.1484];

I^2 = 99.5% [99.5%; 99.6%]; H = 14.89 [14.40; 15.40]

Test of heterogeneity:

Q d.f. p-value

14193.38 64 0

Details on meta-analytical method:

- Inverse variance method

- DerSimonian-Laird estimator for tau^2

- Jackson method for confidence interval of tau^2 and tau

- Log transformation

Appendix H. Article-level and summary results for monthly enrollment per for ARDS/ALI trials only

Number of studies combined: k = 23

rate 95%-CI

Fixed effect model 0.9638 [0.8844; 1.0503]

Random effects model 0.4760 [0.3234; 0.7005]

Quantifying heterogeneity:

tau^2 = 0.8068 [0.5096; 2.0233]; tau = 0.8982 [0.7138; 1.4224];

I^2 = 94.6% [93.0%; 95.8%]; H = 4.30 [3.77; 4.90]

Test of heterogeneity:

Q d.f. p-value

406.64 22 < 0.0001

Details on meta-analytical method:

- Inverse variance method

- DerSimonian-Laird estimator for tau^2

- Jackson method for confidence interval of tau^2 and tau

- Log transformation

Appendix I. Article-level and summary results for monthly enrollment per site by patient population (Sepsis vs. ARDS/ALI)

Number of studies combined: k = 88

rate 95%-CI

Fixed effect model 6.0211 [5.8540; 6.1930]

Random effects model 0.8042 [0.5380; 1.2021]

Quantifying heterogeneity:

tau^2 = 3.6341 [1.9673; 4.0991]; tau = 1.9063 [1.4026; 2.0246];

I^2 = 99.5% [99.4%; 99.5%]; H = 13.79 [13.38; 14.22]

Quantifying residual heterogeneity:

I^2 = 99.4% [99.4%; 99.4%]; H = 13.03 [12.62; 13.45]

Test of heterogeneity:

Q d.f. p-value

16553.93 87 0

Results for subgroups (fixed effect model):

k rate 95%-CI Q I^2

ARDS 23 0.9638 [0.8844; 1.0503] 406.64 94.6%

SEPSIS 65 7.5021 [7.2819; 7.7289] 14193.38 99.5%

Test for subgroup differences (fixed effect model):

Q d.f. p-value

Between groups 1953.90 1 0

Within groups 14600.03 86 0

Results for subgroups (random effects model):

k rate 95%-CI tau^2 tau

ARDS 23 0.4760 [0.3234; 0.7005] 0.8068 0.8982

SEPSIS 65 0.9830 [0.6193; 1.5601] 3.5514 1.8845

Test for subgroup differences (random effects model):

Q d.f. p-value

Between groups 5.57 1 0.0183

Details on meta-analytical method:

- Inverse variance method

- DerSimonian-Laird estimator for tau^2

- Jackson method for confidence interval of tau^2 and tau

- Log transformation

Appendix J. Article-level and summary results for monthly enrollment per site by time period (2009-2013 vs. 2014-2019)

Number of studies combined: k = 92

rate 95%-CI

Fixed effect model 5.8305 [5.6752; 5.9900]

Random effects model 0.8306 [0.5687; 1.2130]

Quantifying heterogeneity:

tau^2 = 3.3690 [1.9076; 3.9063]; tau = 1.8355 [1.3812; 1.9764];

I^2 = 99.5% [99.4%; 99.5%]; H = 13.59 [13.19; 14.01]

Quantifying residual heterogeneity:

I^2 = 99.4% [99.4%; 99.4%]; H = 12.89 [12.50; 13.31]

Test of heterogeneity:

Q d.f. p-value

16818.05 91 0

Results for subgroups (fixed effect model):

k rate 95%-CI Q I^2

2009-2013 42 2.2564 [2.1443; 2.3744] 2304.80 98.2%

2014-2019 50 8.4447 [8.1802; 8.7178] 12659.32 99.6%

Test for subgroup differences (fixed effect model):

Q d.f. p-value

Between groups 1853.93 1 0

Within groups 14964.12 90 0

Results for subgroups (random effects model):

k rate 95%-CI tau^2 tau

2009-2013 42 0.6153 [0.4126; 0.9178] 1.6661 1.2908

2014-2019 50 1.0815 [0.6325; 1.8495] 3.6951 1.9223

Test for subgroup differences (random effects model):

Q d.f. p-value

Between groups 2.73 1 0.0985

Details on meta-analytical method:

- Inverse variance method

- DerSimonian-Laird estimator for tau^2

- Jackson method for confidence interval of tau^2 and tau

- Log transformation

Appendix K. Article-level and summary results for monthly enrollment per site by funding sources (industry vs. non- industry)

Number of studies combined: k = 83

rate 95%-CI

Fixed effect model 6.0194 [5.8437; 6.2003]

Random effects model 0.7643 [0.4961; 1.1774]

Quantifying heterogeneity:

tau^2 = 3.9669 [2.0008; 4.2542]; tau = 1.9917 [1.4145; 2.0626];

I^2 = 99.5% [99.5%; 99.5%]; H = 14.03 [13.60; 14.47]

Quantifying residual heterogeneity:

I^2 = 99.4% [99.4%; 99.5%]; H = 13.39 [12.96; 13.83]

Test of heterogeneity:

Q d.f. p-value

16141.80 82 0

Results for subgroups (fixed effect model):

k rate 95%-CI Q I^2

Industry 19 0.8082 [0.7297; 0.8951] 285.57 93.7%

Non-industry 64 7.2383 [7.0177; 7.4659] 14234.96 99.6%

Test for subgroup differences (fixed effect model):

Q d.f. p-value

Between groups 1621.27 1 0

Within groups 14520.53 81 0

Results for subgroups (random effects model):

k rate 95%-CI tau^2 tau

Industry 19 0.4155 [0.2715; 0.6358] 0.7990 0.8939

Non-industry 64 0.9324 [0.5734; 1.5161] 3.8796 1.9697

Test for subgroup differences (random effects model):

Q d.f. p-value

Between groups 6.01 1 0.0142

Details on meta-analytical method:

- Inverse variance method

- DerSimonian-Laird estimator for tau^2

- Jackson method for confidence interval of tau^2 and tau

- Log transformation

Appendix L. Article-level and summary results for monthly enrollment per site by intervention (drug vs. non-drug)

Number of studies combined: k = 92

rate 95%-CI

Fixed effect model 5.8305 [5.6752; 5.9900]

Random effects model 0.8306 [0.5687; 1.2130]

Quantifying heterogeneity:

tau^2 = 3.3690 [1.9076; 3.9063]; tau = 1.8355 [1.3812; 1.9764];

I^2 = 99.5% [99.4%; 99.5%]; H = 13.59 [13.19; 14.01]

Quantifying residual heterogeneity:

I^2 = 99.4% [99.4%; 99.5%]; H = 13.38 [12.97; 13.79]

Test of heterogeneity:

Q d.f. p-value

16818.05 91 0

Results for subgroups (fixed effect model):

k rate 95%-CI Q I^2

Drug 48 8.1040 [7.8155; 8.4032] 11601.72 99.6%

Non-drug 44 3.8714 [3.7180; 4.0312] 4505.54 99.0%

Test for subgroup differences (fixed effect model):

Q d.f. p-value

Between groups 710.79 1 < 0.0001

Within groups 16107.26 90 0

Results for subgroups (random effects model):

k rate 95%-CI tau^2 tau

Drug 48 0.8148 [0.4412; 1.5047] 4.6311 2.1520

Non-drug 44 0.8530 [0.5562; 1.3082] 2.0355 1.4267

Test for subgroup differences (random effects model):

Q d.f. p-value

Between groups 0.01 1 0.9044

Details on meta-analytical method:

- Inverse variance method

- DerSimonian-Laird estimator for tau^2

- Jackson method for confidence interval of tau^2 and tau

- Log transformation

Appendix M. Article-level and summary results for monthly enrollment per site by continent

Number of studies combined: k = 92

rate 95%-CI

Fixed effect model 5.8305 [5.6752; 5.9900]

Random effects model 0.8306 [0.5687; 1.2130]

Quantifying heterogeneity:

tau^2 = 3.3690 [1.9076; 3.9063]; tau = 1.8355 [1.3812; 1.9764];

I^2 = 99.5% [99.4%; 99.5%]; H = 13.59 [13.19; 14.01]

Quantifying residual heterogeneity:

I^2 = 99.1% [99.0%; 99.2%]; H = 10.62 [10.24; 11.01]

Test of heterogeneity:

Q d.f. p-value

16818.05 91 0

Results for subgroups (fixed effect model):

k rate 95%-CI Q I^2

Europe 45 1.0870 [ 1.0287; 1.1486] 931.05 95.3%

Asia 15 18.0247 [17.2905; 18.7901] 6012.65 99.8%

North America 20 3.8610 [ 3.6005; 4.1402] 1973.99 99.0%

South America 8 7.2873 [ 6.8154; 7.7920] 857.65 99.2%

Australia 4 1.0004 [ 0.8480; 1.1803] 29.58 89.9%

Test for subgroup differences (fixed effect model):

Q d.f. p-value

Between groups 7013.13 4 0

Within groups 9804.92 87 0

Results for subgroups (random effects model):

k rate 95%-CI tau^2 tau

Europe 45 0.5739 [0.4413; 0.7463] 0.7364 0.8581

Asia 15 1.8884 [0.7174; 4.9708] 3.6104 1.9001

North America 20 0.8232 [0.3888; 1.7430] 2.8579 1.6905

South America 8 1.8449 [0.8058; 4.2235] 1.3909 1.1794

Australia 4 0.9461 [0.5542; 1.6151] 0.2650 0.5148

Test for subgroup differences (random effects model):

Q d.f. p-value

Between groups 12.74 4 0.0126

Details on meta-analytical method:

- Inverse variance method

- DerSimonian-Laird estimator for tau^2

- Jackson method for confidence interval of tau^2 and tau

- Log transformation

Appendix N. Article-level and summary results for monthly enrollment per site by consent type (prospective vs retrospective)

Number of studies combined: k = 92

rate 95%-CI

Fixed effect model 5.8305 [5.6752; 5.9900]

Random effects model 0.8306 [0.5687; 1.2130]

Quantifying heterogeneity:

tau^2 = 3.3690 [1.9076; 3.9063]; tau = 1.8355 [1.3812; 1.9764];

I^2 = 99.5% [99.4%; 99.5%]; H = 13.59 [13.19; 14.01]

Quantifying residual heterogeneity:

I^2 = 99.4% [99.4%; 99.4%]; H = 12.87 [12.47; 13.28]

Test of heterogeneity:

Q d.f. p-value

16818.05 91 0

Results for subgroups (fixed effect model):

k rate 95%-CI Q I^2

Prospective 74 7.0386 [6.8423; 7.2404] 14699.64 99.5%

Retrospective 18 0.8411 [0.7682; 0.9209] 196.40 91.3%

Test for subgroup differences (fixed effect model):

Q d.f. p-value

Between groups 1922.02 1 0

Within groups 14896.03 90 0

Results for subgroups (random effects model):

k rate 95%-CI tau^2 tau

Prospective 74 0.8883 [0.5858; 1.3472] 3.2713 1.8087

Retrospective 18 0.6525 [0.4762; 0.8942] 0.4141 0.6435

Test for subgroup differences (random effects model):

Q d.f. p-value

Between groups 1.34 1 0.2469

Details on meta-analytical method:

- Inverse variance method

- DerSimonian-Laird estimator for tau^2

- Jackson method for confidence interval of tau^2 and tau

- Log transformation

Appendix O. Article-level and summary results for monthly enrollment per site by satisfactory enrollment percentage

Number of studies combined: k = 88

rate 95%-CI

Fixed effect model 5.9985 [5.8370; 6.1645]

Random effects model 0.8237 [0.5581; 1.2156]

Quantifying heterogeneity:

tau^2 = 3.4041 [1.9393; 4.0440]; tau = 1.8450 [1.3926; 2.0110];

I^2 = 99.5% [99.4%; 99.5%]; H = 13.80 [13.39; 14.23]

Quantifying residual heterogeneity:

I^2 = 99.4% [99.4%; 99.5%]; H = 13.12 [12.71; 13.54]

Test of heterogeneity:

Q d.f. p-value

16573.54 87 0

Results for subgroups (fixed effect model):

k rate 95%-CI Q I^2

yes 54 8.2407 [7.9889; 8.5004] 13059.43 99.6%

no 34 2.0224 [1.9096; 2.1419] 1733.89 98.1%

Test for subgroup differences (fixed effect model):

Q d.f. p-value

Between groups 1780.22 1 0

Within groups 14793.32 86 0

Results for subgroups (random effects model):

k rate 95%-CI tau^2 tau

yes 54 1.0314 [0.6195; 1.7172] 3.6031 1.8982

no 34 0.5900 [0.3827; 0.9095] 1.5680 1.2522

Test for subgroup differences (random effects model):

Q d.f. p-value

Between groups 2.68 1 0.1016

Details on meta-analytical method:

- Inverse variance method

- DerSimonian-Laird estimator for tau^2

- Jackson method for confidence interval of tau^2 and tau

- Log transformation

Appendix P. Article-level and summary results for monthly enrollment per site by single center vs multicenter

Number of studies combined: k = 92

rate 95%-CI

Fixed effect model 5.8305 [5.6752; 5.9900]

Random effects model 0.8306 [0.5687; 1.2130]

Quantifying heterogeneity:

tau^2 = 3.3690 [1.9076; 3.9063]; tau = 1.8355 [1.3812; 1.9764];

I^2 = 99.5% [99.4%; 99.5%]; H = 13.59 [13.19; 14.01]

Quantifying residual heterogeneity:

I^2 = 99.0% [98.9%; 99.1%]; H = 9.91 [9.55; 10.29]

Test of heterogeneity:

Q d.f. p-value

16818.05 91 0

Results for subgroups (fixed effect model):

k rate 95%-CI Q I^2

1-ICU 20 14.2313 [13.7645; 14.7139] 7040.76 99.7%

>1-ICU 72 1.0700 [ 1.0219; 1.1203] 1801.34 96.1%

Test for subgroup differences (fixed effect model):

Q d.f. p-value

Between groups 7975.95 1 0

Within groups 8842.10 90 0

Results for subgroups (random effects model):

k rate 95%-CI tau^2 tau

1-ICU 20 4.8616 [2.4856; 9.5090] 2.3301 1.5265

>1-ICU 72 0.5219 [0.4115; 0.6618] 0.9804 0.9902

Test for subgroup differences (random effects model):

Q d.f. p-value

Between groups 37.77 1 < 0.0001

Details on meta-analytical method:

- Inverse variance method

- DerSimonian-Laird estimator for tau^2

- Jackson method for confidence interval of tau^2 and tau

- Log transformation

Appendix Q. Characteristics of studies which stopped early citing enrollment difficulties (n=8)

| ID | Syndrome | Continent | Sites | Funding | Intervention | Consent | Duration of Enrollment (months) | Enrollment Rate | Number of patients randomized | Target enrollment | Percent of Target Enrollment Achieved |
| --- | --- | --- | --- | --- | --- | --- | --- | --- | --- | --- | --- |
| 20 | Sepsis | North America | 7 | Government | Device | Prospective | 48 | 0.11 | 37 | 120 | 31% |
| 25 | Sepsis | Europe | 64 | Industry | Drug | Prospective | 36 | 0.09 | 199 | 270 | 74% |
| 29 | Sepsis | Asia | 13 | Foundation | Drug | Prospective | 46 | 0.10 | 60 | 200 | 30% |
| 41 | Sepsis | Europe | 18 | Government | Device | Retrospective | 53 | 0.15 | 140 | 460 | 30% |
| 56 | ARDS | North America | 3 | Government | Drug | Prospective | 56 | 0.79 | 132 | 200 | 66% |
| 61 | Sepsis | Europe | 12 | Government | Device | Prospective | 36 | 0.18 | 76 | 400 | 19% |
| 75 | Sepsis | Asia | 1 | Government | Drug | Prospective | 58 | 1.33 | 77 | 150 | 51% |
| 92 | ARDS | Asia | 10 | Foundation | Protocol | Prospective | 40 | 0.10 | 40 | 90 | 44% |
